# Prevalence of the use of prescription stimulants as “study drugs” by UK university students: A brief report

**DOI:** 10.1002/brb3.3419

**Published:** 2024-02-12

**Authors:** Ffinian Jones, Philip M. Newton

**Affiliations:** ^1^ Swansea University Medical School Swansea University Swansea UK

**Keywords:** academic integrity, addiction, cosmetic neurology, neuroenhancement, prescription stimulants

## Abstract

**Introduction:**

There is media concern over students using prescription stimulants as “cognitive enhancers” to try and improve their academic performance. However, there is limited evidence about the prevalence of this behaviour in the United Kingdom, or whether it has increased in recent years.

**Methods:**

We review survey studies on students' use of cognitive enhancers.

**Results:**

Overall reported use is low, with some inconclusive evidence that it is increasing. Use of modafinil appears to be higher than that of methylphenidate or dexamphetamine.

**Conclusion:**

There is a clear need for large‐scale research in this area, using representative sampling and survey methods that protect student anonymity.

## Graphical abstract

We investigated the number of students in the United Kingdom using certain stimulant medicines, without a prescription, as “study drugs” to improve their performance in assessments. There is concern that these drugs can have harmful side effects and may be considered cheating. We found that a small minority of students are using these drugs, although one (modafinil) is more common. Most importantly, we found that there needs to be rigorous high‐quality research to truly understand how many students are using these medicines and the possible harmful effects.

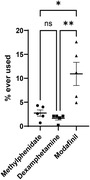

## 1 INTRODUCTION

Cognitive enhancement is defined as “the use of medications or other brain treatments for improving normal healthy cognition” (Farah, 2015). Three prescription drugs are cited as being commonly used, without a prescription, for their cognitive enhancing effects—modafinil, methylphenidate, and dexamphetamine—and the use, legal status, and availability of these drugs varies by country (Esposito et al., 2021; Sharif et al., 2021). Data on the prevalence of the use of these drugs for cognitive enhancing effects in the United Kingdom are currently lacking.

Modafinil is prescribed in the United Kingdom as Provigil for the treatment of narcolepsy (NICE, 2023). In all users, it promotes a heightened state of wakefulness through a complex and poorly understood modification of multiple neurochemical pathways including inhibition of dopamine and noradrenaline reuptake transporters (Madras et al., 2006). A small number of studies show improvements in cognitive function with modafinil use, which are enhanced in sleep‐deprived individuals (Kredlow et al., 2019). Modafinil appears to have limited abuse potential and is well tolerated, although common side effects include anxiety, irregular heartbeat, headache, insomnia, nausea, and dizziness (Hashemian & Farhadi, 2020). Modafinil is currently a Schedule IV(II) substance in the United Kingdom, meaning that it is illegal to supply without a prescription, but not illegal to possess (DrugScience UK, 2023). It is freely available via the black market for less than 0.5 GBP (∼0.65 USD) per dose (Hockenhull et al., 2020).

Methylphenidate, (UK trade name Ritalin) and dexamphetamine (UK trade name Adderall) are used for the treatment of attention deficit hyperactivity disorder (ADHD). They block catecholamine transporters (Fleckenstein et al., 2007), but the evidence supporting their effectiveness as cognitive enhancers (CEs) in individuals without ADHD is modest and domain specific (Roberts et al., 2020). Both have more serious side effects than modafinil and the potential for addiction, especially amphetamine (Steinkellner et al., 2011).

An analysis of media coverage of cognitive enhancement showed that it is widely portrayed as “common” and/or “increasing,” but there are limited data to support this claim, particularly in the United Kingdom (Partridge et al., 2011) where there is currently a focus on illicit drug use by university students, aimed at developing policies and strategies to reduce harm (Universities UK, 2022). Recent systematic reviews gave general overviews of CE use in universities worldwide; however, they did not quantify the overall levels of use (Esposito et al., 2021; Sharif et al., 2021).

Thus, our research questions were to test (1) how common is it for university students in the United Kingdom to use these three medicines, without a prescription, for cognitive enhancement, and (2) whether use is increasing.

## 2 METHODS

The study is conducted and reported according to the principles of the 2020 version of the PRISMA statement for systematic reviews (Moher et al., 2009). Quality measures were taken from similar studies of challenging topics in education (Newton, 2018; Newton & Essex, 2023; Newton & Salvi, 2020).

### 2.1 Information sources and search strategy

We were fortunate that two recent systematic reviews have been published on the use of CEs by university students, citing studies up to and including 2020 (Esposito et al., 2021; Sharif et al., 2021). We therefore identified studies from these reviews that met the eligibility criteria below. However, these reviews did not quantify the use of CEs and did not include dissertations or other reports. In order to identify PhD theses and other sources, we searched Google Scholar, which has the best coverage of “gray literature” (Haddaway et al., 2015). However, the current functionality of Google Scholar means that it is not possible to quantify our search strategy in a way that would allow us to report a PRISMA flow diagram (e.g., Google Scholar does not allow the exclusion of the search results from prior searches, and so it is not possible to quantify the number of unique search results). However, the majority of search details are reported in the aforecited existing systematic reviews (Esposito et al., 2021; Sharif et al., 2021). Therefore, we repeated the search strategy of the aforecited reviews in order to identify studies published after 2020, along with PhD theses. In addition, we used the following search terms: “modafinil” AND “student” AND “United Kingdom”; “Adderall” AND “student” AND “United Kingdom”; and “prescription stimulants” AND “student” AND “United Kingdom” AND “survey.”

Inclusion criteria were as follows:

- Use of one or more of the three specified drugs as a CE;
- Participants were current students in Higher Education in the United Kingdom (one study was open to students from Ireland [Singh et al., 2014], but the majority of the participants were from the United Kingdom, so this was included);
- Reported sufficient data to allow the calculation of the number of students surveyed, and the number who report taking one or more of the three CEs.

Exclusion criteria were as follows:

- Recreational drug use;
- Use by individuals who were not currently students.

### 2.2 Data items

The following items were identified where possible:

- Year study was published;
- Year research was undertaken;
- Whether ethical approval was reported;
- Whether a conflict‐of‐interest statement was reported;
- The discipline being studied by the student participants;
- Sources of funding;
- The sampling method. This was classified as in similar work on survey‐based research in Higher Education (Newton & Salvi, 2020):
  ○ *Convenience*. Survey distributed to all in a specified population. Participants voluntarily completed the survey, and these responses form the dataset.
  ○ *Snowball*. As for convenience sampling but with further distribution via unregulated sources (e.g., social media) and so no control over who gets invited and when.
  ○ *Unclassifiable*.
- The target population name and size;
- The number of individuals sampled from that population;
- The number who completed the survey (this is reported as “*N*”);
- The response rate (this is *N*/target population number);
- The number of participants reporting ever using any one of the three CEs, expressed as a percentage of “*N*.”

### 2.3 Synthesis and analysis

The total number of students using each individual drug and the total number of students using any drug were calculated across all studies. One study (Heyes, 2022) used three different methods of questioning to answer the research question. The data reported are from the direct questioning method as this best reflects the methods used in other studies and so allows for direct comparison. The other two methods are considered in the discussion. Specific statistical tests are reported in the results.

## 3 RESULTS

### 3.1 Study selection

The aforecited reviews included six studies that fit the eligibility criteria. We identified four additional studies: two PhD theses, a paper from 2016, and a conference abstract from 2014.

### 3.2 Study characteristics and results

Studies and their key characteristics are shown in Table 1. The majority of studies were full studies published in peer‐reviewed journals, although two were PhD theses (Heyes, 2022; Tully, 2020) and one was a conference abstract (Pennington, 2014).

**TABLE 1:** Studies included in the analysis.

Study	Sampling method	Target population	*N*	Response rate (%)	Using methylphenidate (%)	Using dexamphetamine (%)	Using modafinil (%)	Any cognitive enhancer (%)
McDermott et al., 2021	Sno	NS	506	–	4.3	0.2	17.6	18.18
Champagne et al., 2019	Sno	29,600	420	1.42	2.1	1.7	11.2	16.43
Hanna et al., 2018	Con	221	198	89.59	–	–	–	2.5
Holloway & Bennett, 2012	Con	14,839	1517	10.22	–	–	–	0.20
Nguyen et al., 2021	Con	29,600	148	0.50	0.7	2.1	15	17.49
Singh et al., 2014	Con	2,860,000	877	0.03	4.0	2.1	6.2	9.4
Heyes, 2022	Con	2,860,000	722	0.03	–	–	–	3.72
Tully, 2020	Sno	NS	389	–	2.6	2.1	4.9	6.7
Helmer et al., 2016	Con	NS	107	–	–	–	–	11
Pennington, 2014	Unc	NS	113	–	–	–	–	2.65

*Note*: Con = Convenience sampling; Sno = Snowball sampling; Unc = Unclassifiable.

Abbreviation: NS, not stated.

### 3.3 Use of CEs

**FIGURE 1:**
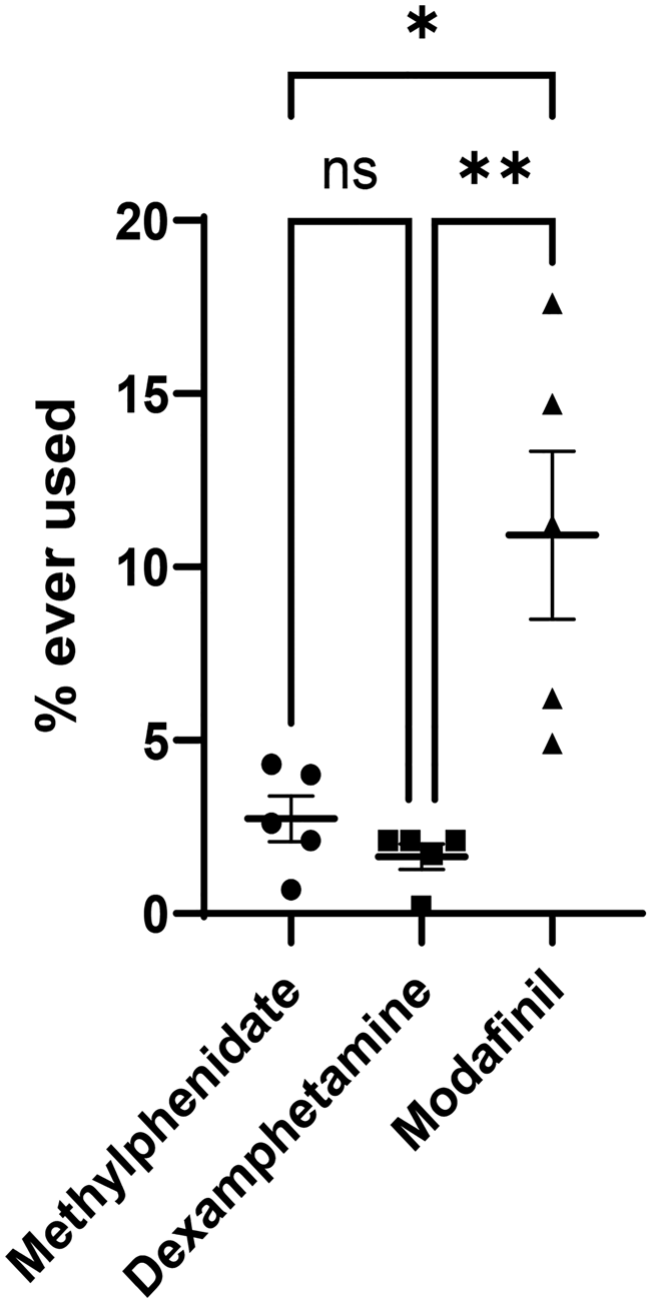
The lifetime use of specific cognitive enhancers by university students in the United Kingdom. Data are shown as a scatter plot with the mean ± standard error.

Lifetime use of CEs was reported by 6.9% (345/4997) participants. Five studies (*N* = 2340) measured use of the three CEs separately. Lifetime use for modafinil (9.9%, 231/2340) was higher than for methylphenidate (3.3%, 77/2340) or dexamphetamine (1.6%, 37/2340). Data were normally distributed according to a Kolmogorov–Smirnov test and so differences between drugs were evaluated using a one‐way repeated measures ANOVA (*F*(2, 8) = 10.5, *p* < .0058). Post hoc Holm–Sidak tests revealed significant differences between modafinil and methylphenidate (*p* = .0121), and between modafinil and dexamphetamine (*p* = .0091), but not between methylphenidate and dexamphetamine (*p* = .6330) (Figure 1).

### 3.4 Change over time

**FIGURE 2:**
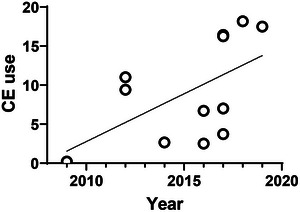
The lifetime use of any cognitive enhancer by university students in the United Kingdom. See results for analysis explanation. CE, cognitive enhancer.

A simple linear regression was conducted to predict CE use based on time. The independent variable was the year the study was conducted. The linear regression model was not significant, although the result was borderline (*F*(1, 10) = 4.526, *p* = .0593), with a suggestion that use was increasing over time. The model explained 31.2% of the variance in CE use (Figure 2).

### 3.5 Response rate

Most studies did not report sufficient information to allow a calculation of response rate. Some studies targeted all students in the United Kingdom; therefore, the total number of students in the United Kingdom (2.86 million [Bolton, 2023]) was used. Two studies targeted all students at Kings College in London; therefore, the total number of students studying at Kings College in London (29,600 [UoL, 2023]) was used.

### 3.6 Sampling method

No studies used any form of representative sampling method.

## 4 DISCUSSION

This Short Report summarizes research on the number of UK university students using prescription‐only stimulants as CEs. We found that overall use is low, with a mean prevalence of 6.9% across all studies. The studies indicated that modafinil use is higher than that of methylphenidate and dexamphetamine, with a suggestion, although not significant, that total use has increased since 2011.

However, perhaps our main finding is the methodological limitations of the studies themselves. Most samples were small and none were collected using a representative sampling method. Convenience or snowball sampling is associated with an underestimation in the prevalence of challenging behaviors in student populations (Newton, 2023). All studies here used a form of direct questioning (e.g., “*have you ever…*”). One study (Heyes, 2022) compared this approach to two forms of indirect questioning that give participants greater confidence in their anonymity. Indirect methods yielded much higher estimates of CE use (3.7% vs. 7% or 16.6%). Thus, the methodological features of the surveys reviewed here make it likely that they underestimate the rate of CE use, suggesting a need for large‐scale research on the subject, using representative sampling methods. This could facilitate analysis of factors that potentially influence the use of CE, such as age, gender, legal status, and efficacy.

Even the apparently low rates of CE use reported here could be considered problematic. There are 2.86 million university students in the United Kingdom (Bolton, 2023). Thus, the data here suggest that 200,000–300,000 of them are using CEs. These are all regulated prescription stimulants whose off‐label use is associated with a range of risks as reviewed in the introduction. The drugs are widely and cheaply available via the black market (Hockenhull et al., 2020), and students view CEs as safer than recreational drugs due to their prescription status (Looby et al., 2014); however, it is debatable how much students are aware of the risks involved (de Souza, 2015).

Then, there is a further question regarding the ethics of CE use. It remains an open question whether or not the use of these CEs by University students constitutes “cheating,” with compelling arguments both for and against in the literature (Inon, 2019; Porsdam Mann et al., 2018). From a pragmatic perspective, UK universities do not even consider the issue in their policies on Academic Integrity, or alcohol and drug misuse (Heyes, 2022), even though they are on the World Anti‐Doping Agency list of banned substances and so are prohibited for use by athletes including those participating in diverse competitions such as chess and competitive video gaming, and competitions will screen athletes for their use of CEs (Dance, 2016).

In summary, the current use of CEs by UK university students is unclear, but it is likely to be low. Much more research is needed to fully understand whether this issue represents a risk to student health, and to academic standards in UK Higher Education.

## AUTHOR CONTRIBUTIONS

**Ffinian Jones**: Investigation; methodology; writing—original draft. **Philip Newton**: Conceptualization; data curation; formal analysis; investigation; methodology; project administration; supervision; visualization; writing—original draft; writing—review and editing.

## CONFLICT OF INTEREST STATEMENT

The authors declare no conflicts of interest.

### FUNDING INFORMATION

This work was not supported by any funding body.

### PEER REVIEW

The peer review history for this article is available at https://publons.com/publon/10.1002/brb3.3419.

## Data Availability

Data sharing is not applicable to this article as no new data were created or analyzed in this study.
